# Paternalism as Punishment

**DOI:** 10.1017/S0953820820000254

**Published:** 2020-09-21

**Authors:** David Birks

**Affiliations:** University of Hong Kong, University of Oxford

## Abstract

In this article, I argue that even if we hold that at least some paternalistic behaviour is impermissible when directed towards innocent persons, in certain cases, the same behaviour is permissible when directed towards criminal offenders. I also defend the claim that in some cases it is morally preferable to behave paternalistically towards offenders as an alternative to traditional methods of punishment. I propose that the reason paternalistic behaviour is sometimes permissible towards an offender is the same reason that inflicting intentional harm on an offender is permissible – namely, that it is sometimes a morally justified method of punishing an offender for his wrongdoing.

If it is impermissible to prevent an innocent person from harming himself, is it impermissible to prevent a criminal offender from doing the same? Similarly, if it is impermissible to compel an innocent person to pursue activities that benefit him, is it also impermissible to compel the same from a criminal offender? Both questions concern the permissibility of paternalism, which is generally thought to be morally objectionable, regardless of whether a person has committed a criminal offence.

In this article, I argue that even if we hold that at least some paternalistic behaviour is impermissible when directed towards innocent persons, in certain cases, the same behaviour is permissible when directed towards criminal offenders.^1^ Rather than deploy traditional punishments, which are often significantly harmful for many offenders, I defend the claim that in some cases it is morally preferable to impose a benefit through paternalistic behaviour. I propose that the reason paternalistic behaviour is sometimes permissible towards an offender is the same reason that inflicting intentional harm on an offender is permissible – namely, that it is sometimes a morally justified method of punishing an offender for his wrongdoing. Let’s call paternalistic behaviour that is deployed as punishment, *paternalistic punishment.*

There are two types of paternalistic punishment. The first type is punishment that *denies* the offender something with the intention of benefitting him. For example, we could deny the offender cigarettes on the grounds that smoking is bad for his health. The second type *compels* the offender to act in some way with the intention of benefitting him. So, there might be some valuable activities that the offender does not want to do, and we could intend to benefit the offender by compelling him to participate in these activities. For instance, the offender could be compelled to exercise, or to read works of valuable literature.^2^

The reason to impose these benefits as punishment is not necessarily to rehabilitate the offender, although as I will go on to show, they could be imposed for this purported reason. Rather, I propose that we could deploy paternalistic punishment as a method of acting in accordance with other commonly held reasons why we should punish, such as retribution or deterrence. It could be deployed either as an alternative to traditional punishment, or to supplement a reduced sentence of traditional punishment. Indeed, the paternalistic benefits do not need to connect to the wrongness of the offence. We could deny tobacco as a punishment for offences unrelated to smoking, such as vandalism. Or we could compel an offender to exercise, as a punishment for tax fraud.^3^

Paternalistic punishment might seem strikingly objectionable on several grounds. First off, it might seem simply a mistake to call it punishment. Punishment is intended to be harmful, while paternalistic behaviour is intended to be beneficial, and it might be inconceivable that the latter could be a means of imposing the former. But even if an act of paternalism could be punishment, it might also seem objectionable on the grounds that we would not achieve the purported objectives of punishment by deploying it. For instance, it might be thought that those who hold we are justified in punishing offenders for retributive or deterrence reasons would not be satisfied by this method of punishment. How could a beneficial punishment treat the offender as he deserves, or deter offending?

Finally, even if these concerns were adequately addressed, paternalism might still be objectionable as a method of punishment. After all, paternalistic behaviour towards informed competent adults is commonly thought to be a significant wrong, and it is not obvious why the fact a person has committed an offence eliminates this fact.

In what follows, I show that these objections to paternalistic punishment are misplaced. In fact, it will become clear that not only can these concerns be addressed, but in some cases there are grounds to prefer paternalistic punishment to traditional forms of punishment.

I begin in Section 1 by setting out how paternalistic behaviour can constitute punishment. Then, in Section 2, I seek to establish that paternalistic punishment is sometimes a permissible method of punishment. In order to do this, I consider how *any* form of punishment could be permissible. Theories of punishment are attempts to point out reasons that justify punishment. While we usually have reasons to refrain from harming others, theories of punishment claim that goals such as deterrence or retribution sometimes give us reasons to punish that defeat the reasons not to inflict harm. I argue that paternalistic punishment can be one method of acting in accordance with these reasons to punish. Following this, in Section 3, I provide three grounds for holding paternalistic punishment to be a morally preferable method of administering punishment. Next, in Section 4, I consider a serious objection to paternalistic punishment, namely that paternalistic behaviour is a significant wrong in a respect that traditional punishment is not. Therefore, even if paternalistic punishment has several advantages over traditional punishment, it might still be impermissible. Nevertheless, I argue, the fact that punishment is paternalistic does not necessarily render it impermissible.

Before I proceed to the main arguments of the article, I should make an important clarification. The idea that we ought to benefit offenders as a response to their wrongdoing has a long philosophical tradition, including Plato, Kierkegaard, and Hegel.^4^ It also has some contemporary proponents, such as Jean Hampton and Herbert Morris.^5^ My proposal of paternalistic punishment is very different from these traditional paternalistic *theories* of punishment.

The crucial difference is that paternalistic theories of punishment are providing an answer to the question, *why* should we punish offenders? They answer that the justifying reason to punish offenders is to confer a specific benefit, namely, the benefit of moral improvement. My account is a response to a different question, namely, *how* should we punish offenders? I propose that, regardless of whether one thinks the justification of why we should punish is for retributive, deterrence, or rehabilitative reasons, we can act in accordance with those reasons by administering paternalistic punishment (at least in some cases). Indeed, paternalistic punishment can be morally preferable as an alternative to traditional punishments, while achieving exactly the same retributive, deterrent, or rehabilitative ends.

This clarification is particularly important, as paternalistic theories of punishment face serious objections.^6^ My argument is that one can reject paternalistic theories of punishment, and hold a different justification of punishment, such as retributivism, or deterrence, but still accept paternalistic behaviour as a method of punishing offenders.

## 1 How can paternalistic behaviour constitute punishment?

Recall the examples above where, as a response to an offence, we deny the offender cigarettes on the grounds that smoking is bad for his health, or where we compel an offender to read works of valuable literature to benefit him. In this section, I explain how both are instances of paternalistic behaviour and can constitute punishment.

According to a plausible definition of paternalism, an agent behaves paternalistically when he intends to benefit another person while being motivated by a negative judgement of the beneficiary’s ability (assuming he has the relevant information) to make the right decision or manage the particular situation, in a way that will effectively advance his wellbeing in some respect.^7^ So, for instance, suppose we prevent an offender from smoking, in order to protect his health. Here, we are acting paternalistically because we intend to benefit the offender by protecting his health, and we are motivated to intervene in his actions either by a negative assessment of his judgement about the merits of smoking, or by a negative assessment of his willpower to follow through on his judgement that he should not smoke. Similarly, in the case of requiring the offender to participate in a valuable activity, we require him to do it with the intention he will benefit from it; and we have the negative judgement that without the compulsion he is less likely to do it.

These instances of paternalistic behaviour can be punishment. For the purposes of the article, I accept that we punish an alleged offender when and only when we inflict intentional harm on him as a response to his alleged wrongdoing, while expressing disapproval of that alleged wrongdoing.^8^ The most contentious aspect concerns the *harm requirement*, that is, the requirement that in order for behaviour to be punishment, intentional harm needs to be inflicted, rather than pain,^9^ burdens,^10^ a loss in some respect,^11^ or a diminution of liberty.^12^ I stipulate a definition with this requirement because it is the one least obviously compatible with paternalism. While it is uncontroversial to hold that many forms of paternalistic behaviour inflict pain, burdens, a loss in some respect, or a diminution of liberty, it is less clear that they could be harmful. After all, paternalistic behaviour is intended to benefit the paternalisee. So, it might seem as though what is inflicted on the offender can either be punishment, or it could be paternalistic. But it cannot be both.

We can circumvent this worry if we unpack the harm requirement. First, in order for it to be plausible, punishment needs to inflict only a *pro tanto* harm on the offender. It does not need to inflict all things considered harm. I suspect that a definition of punishment that required an all things considered harm would not be shared by many. To see this, imagine a person, call her Victoria, who spends her life committing petty crime, such as stealing from loved ones and strangers.^13^ Victoria is eventually caught stealing, and she is forced to spend a period in prison. During this time in prison, she reflects on her previous life of wrongdoing, and commits to devoting her life to valuable projects. She then proceeds to live a fulfilling life helping others. It seems plausible to think that Victoria’s life has gone better as a consequence of her incarceration. If we hold that a necessary condition of punishment is that it needs to be all things considered harmful, then it would be the case that Victoria was not punished for her offence. But this seems a peculiar understanding of punishment far removed from its everyday usage.

Alternatively, if we hold that the harm requirement needs only that *pro tanto* harm is inflicted, this is compatible with the judgement that Victoria was punished. There could be many *pro tanto* harms inflicted on Victoria, such as the harms of being imprisoned, and the frustration of her autonomous pursuits. Whether or not these harms eventually resulted in Victoria benefitting, it seems plausible to hold that she has been punished for her offence.

It might be objected that even once we clarify the harm requirement, there remains a concern about the possibility of intending to inflict both a harm and a benefit on the offender. There might seem something contradictory about intending to harm and intending to benefit the same person with an action. While I am unable to provide an analysis of the nature of intentions here, it is worth noting that it is not an unusual thought that we can perform actions with multiple intentions. When we perform the action of getting out of bed, we might have two intentions, for example: the intention to eat breakfast and the intention to meet a friend. Moreover, intentions can have a hierarchical structure, with intentions nested within other intentions.^14^ For instance, we might have the general intention to punish the offender, and so intend to inflict *pro tanto* harm on him. But nested within that general intention is the specific intention to behave paternalistically, that is, to intend to make the offender all things considered better off. The person imposing the punishment believes he is only justified in selecting from among possible actions that inflict *pro tanto* harm on the offender. However, of those options, he intends to inflict a *pro tanto* harm that will result in the offender being all things considered better off. The paternalistic intention is nested within the punishment intention. The punishing agent has a pro attitude to both features and they are both part of his reason for imposing the paternalistic punishment, rather than imposing a non-paternalistic punishment.

We might now ask, in what respect is paternalistic behaviour *pro tanto* harmful? While I am unable to provide a catalogue of the kinds of harms inflicted by some paternalistic behaviour, I will briefly mention three possible harms.

One might hold that paternalistic behaviour inflicts *pro tanto* harm when it involves an interference with a person’s autonomous projects and relationships, regardless of whether the paternalisee is aware that the interference has taken place. For example, suppose I believe that you would have a better life if you changed your career from political philosophy to professional tap-dancing. Consequently, without you knowing, I hypnotise you so that you change your career. If the hypnotism was contrary to your autonomous project to pursue political philosophy, one might hold that you have been harmed in one respect, even if you proceed to have a very happy and successful tap-dancing career.

One might also think that paternalistic behaviour is harmful because it causes two types of *experiential* harm. One type is the negative experience of feeling disrespected and infantilised when one knows that one is being paternalised. A further type could occur as a consequence of being denied something, or being compelled to do something. For example, if we deny tobacco to an offender who regularly consumes it, he will experience unpleasant withdrawal symptoms. If we compel an offender to exercise, he might find it painful and tiring. These experiences might be thought to be harmful.

Finally, one might think that paternalistic punishment involves harm due to the method of its enforcement. Suppose we deny an offender tobacco as a response to his wrongdoing. In order to ensure that the offender complies we might require him to attend regular tests, and those tests might be harmful. For example, it is sometimes painful to have blood tests. It is also inconvenient to attend regular appointments, and can cause disruption to one’s career, or other projects. The method of enforcing the paternalistic punishment could involve both experiential harms and interference with the offender’s autonomous projects.

How can paternalistic behaviour express condemnation of wrongdoing? The fact that we intentionally inflict harm as a response to an alleged offence is one ground for holding that paternalistic punishment expresses condemnation.^15^ But it is worth noting that it also expresses condemnation precisely because it involves treating offenders in a way that would be commonly thought to be impermissible when directed towards innocent persons, for reasons unrelated to the harm inflicted. Many forms of paternalistic behaviour are thought to be impermissible regardless of whether they are harmful. There is a vast literature defending this view, and it is reflected in law in many jurisdictions.^16^ The wrongness of paternalism does not solely depend on its harmfulness. The fact that, as a response to wrongdoing, we treat the offender in a way that is thought to be impermissible if applied to innocent persons in itself contributes to an expression of disapproval of his wrongdoing. It does so, first, by creating a distinction between our treatment of offenders and innocent persons, and then, by treating offenders in a way we believe to be worse, in at least one respect.

In summary, many instances of paternalistic behaviour can constitute punishment. They can meet the harm requirement, and express disapproval of wrongdoing.

## 2 Paternalism and the reasons to punish

In the previous section, I have shown that paternalistic behaviour can constitute punishment. I have not yet established that paternalistic punishment is permissible punishment. In order to do this, I need to show two things. First, I need to show that paternalistic punishment enables us to achieve the objectives of a variety of different theories of punishment, namely, rehabilitation, retributivism, and deterrence. For if we hold that it is permissible to punish on the basis of one of these reasons, and we can act in accordance with one of these reasons by deploying paternalistic punishment, this means that it is at least prima facie eligible as a possible punishment. However, this by itself does not mean that paternalistic punishment could be preferable to traditional punishment, nor that it is permissible. For even if we can act in accordance with one of the reasons to punish by administering paternalistic punishment, there could be further reasons specific to the wrongness of paternalism that mean that it is an impermissible form of punishment. So, my second task is to demonstrate that even if one holds paternalism to be a significant wrong, this does not necessarily mean that paternalistic punishment is impermissible. In this section, I focus on the first task, and I turn to the second task in Section 4. The third task, to defend the view that paternalistic punishment is in some cases morally preferable, will be addressed in Section 3.

Let’s begin by discussing how any method of punishment can be permissible. After all, punishment is by definition harmful, and intentionally inflicting harm on innocent persons is typically impermissible. What makes it permissible to inflict intentional harm on offenders but not on innocent persons (at least in most cases)? In order for it to be at least permissible to punish, there needs to be a reason that counts in favour of punishing offenders that defeats the reasons not to inflict intentional harm. Theories of punishment, such as retributivism and deterrence, are attempts to point out such reasons. According to retributivists the reason to punish is generated by the reason to treat offenders as they deserve. Similar thinking applies in the case of deterrence. The reason to deter further offences is held to defeat the reason not to inflict harm.

The question is, then, how do we act in accordance with these reasons to punish? Typically, we do so by deploying traditional punishments. These include financial penalties, community service, and for more serious offences, incarceration. But it is worth noting that if deployed with the relevant intentions and motives, any punishment could be, in principle, a paternalistic punishment. Indeed, some traditional punishments could be, for some offenders, beneficial. For instance, it is possible that an offender might benefit from spending a period alone to reflect on his wrongdoing. Paternalistically imposing incarceration could be one way of conferring this benefit.

Nevertheless, given that prevailing prison conditions expose inmates to risk of significant harm, including sexual assault and other violence, incarceration as it is typically practised would not be a beneficial paternalistic punishment for most offenders.^17^ In fact, all traditional punishments are likely to be harmful. So, for the sake of simplicity, in what follows, when I contrast traditional punishments with paternalistic punishments, I assume the former are all things considered harmful for the offender, and the latter are all things considered beneficial. I do not defend paternalistic punishment where the agent intends to benefit the offender, but in fact fails to do so. My aim in this section is to show that paternalistic punishment is one further method of acting in accordance with the reasons to punish, and hence, paternalistic punishment is a viable alternative to traditional punishment.

Of all the reasons to punish, rehabilitation is the one most obviously compatible with paternalistic punishment.^18^ This is the view that there is a reason to punish in order to morally reform the offender. We could act in accordance with this rehabilitation reason by administering paternalistic punishments, such as compelling a violent offender to attend anger management class, or compelling a drug addict to attend an addiction recovery programme.

Not all paternalistic punishments satisfy this reason. This is because many forms of paternalistic punishment unrelated to an offence do not have any tendency towards rehabilitation. For example, a paternalistic punishment that denied cigarettes to a violent offender would not rehabilitate him.

The use of paternalism in order to rehabilitate is a common part of the criminal justice system in many jurisdictions, and as a result, I do not dwell on this reason. More interesting is the fact that we can act in accordance with retributive and deterrence reasons by deploying paternalistic punishment, and this will be the focus for the remainder of the section.

### 2.1 The retributive reason to punish

Let’s consider two types of retributivism. I call the first type, crude retributivism. On this theory of punishment, there is a significant reason to punish in order to make the offender suffer, because the offender deserves to suffer as a response to his wrongdoing.^19^ I propose that we could act in accordance with this crude retributivist reason by inflicting paternalistic punishment insofar as it caused the offender sufficient suffering in proportion to what he deserves. I will say more about proportionality below. But the fact the offender deserves to suffer, and the paternalistic punishment causes him to suffer, means that we can act in accordance with this reason by administering paternalistic punishment. The fact that the punishment also benefits the offender in addition to inflicting the deserved suffering does not undermine this. The fact that it is all things considered good for the offender does not mean the punishment has failed to give the offender what he deserves.

Nevertheless, a more sophisticated form of retributivism might hold that there is a significant reason to inflict punishment in order to make the offender all things considered worse off, rather than to suffer, because the offender deserves to be made all things considered worse off as a response to his wrongdoing.^20^

Now, I admit that it is difficult to see how paternalistic punishment could be a method of acting in accordance with this reason. But what I will do here is show that the sophisticated retributivist is unlikely to hold this view as stated. Instead he is more likely to hold a slightly different view. This is because, as currently presented, sophisticated retributivism faces a problem. Specifically, it would not permit any form of punishment that resulted in the offender’s life becoming better as a consequence of the punishment. I propose that a more plausible understanding of sophisticated retributivism allows the offender to be all things considered better off following the punishment. As a consequence, paternalistic punishment can be a means of giving offenders what they deserve.

Consider the earlier case of Victoria. According to the sophisticated retributivist, she would not have been sufficiently punished. Imagine at T_1_ we inflict a non-paternalistic punishment on Victoria. At T_2_, as a consequence of the punishment, Victoria changes how she lives her life, and then in fact, her life goes better than it otherwise would have gone had she not been punished. Given that the punishment at T_1_ did not make Victoria all things considered worse off, according to the sophisticated retributivist we did not sufficiently punish her. It might be the case that at T_3_ we should administer punishment again to Victoria to ensure that she is made all things considered worse off and is treated as she deserves. But this seems implausible, and it seems likely that the sophisticated retributivist would not want to bite this bullet.

Now, there are a couple of ways for the sophisticated retributivist to attempt to avoid this conclusion. He could respond by saying that there is a morally relevant difference between cases where the offender is made all things considered better off through paternalistic punishment, and cases like Victoria, where the non-paternalistic punishment results in the offender being all things considered better off by way of some other mechanism. They might appear the same because they both involve offenders who have lives that go better as a consequence of punishment. But the sophisticated retributivist could claim that a morally relevant difference is revealed once we consider the possible benefits imposed in more detail.

One possible relevant difference could be that the punishment in cases like Victoria inflicts all things considered harm at the time of its infliction (a time restriction), whereas paternalistic punishments do not. At T_1_ the punishment inflicted on Victoria makes her all things considered worse off, it restricts her liberty, and causes her numerous experiential harms. Even if the punishment later results in her life going all things considered better than it would have had she not been punished, the sophisticated retributivist might hold that the fact that it made her all things considered worse off at the time of the infliction means that she has been sufficiently punished. The fact the punishment might later result in Victoria being all things considered better off does not prevent the punishment giving her what she deserves.

Let’s accept for the sake of argument that this move is defensible. It means that in cases like Victoria, the offender can be sufficiently punished even if it results in her being all things considered better off, whereas some paternalistic punishments – those that do not inflict all things considered harm at the time of the punishment – would not constitute sufficient punishment. For example, imagine that an offender deserves to be harmed and in order to inflict the harm, we perform a lifesaving surgical procedure on him, despite the fact that he autonomously chose not to have it performed. While we might think that the surgery will be harmful, on the grounds that might include the fact that it infringes the offender’s autonomy, it is still plausibly all things considered good for him at the time it is inflicted and so not all things considered harmful, as required by the account of punishment we are considering. But these types of paternalistic punishments are unusual. Many paternalistic punishments would not be ruled out by a time restriction because often the benefits of paternalism occur long after it takes place. For example, consider the paternalistic punishment of compelling the offender to exercise. We might think that if we compel an offender to exercise strenuously enough, it inflicts an all things considered harm at the time of the punishment. There may be benefits at the time of the punishment, but these are outweighed by the immediate harm caused by the compelled exercise. As the punishment made the offender all things considered worse off at the time it was inflicted, it could constitute sufficient punishment for sophisticated retributivism with a time restriction. However, the primary benefit of being compelled to exercise occurs after the time of the punishment. For example, typical benefits of exercise include a lower risk of cancer, depression, heart disease, and stroke.^21^ Even if there are several *pro tanto* harms intentionally inflicted when we compel an offender to exercise that outweigh any immediate benefits, the fact that the offender is more likely to live a longer and healthier life as a consequence of the punishment would mean that it is an all things considered benefit to the offender. But this meets the time restriction since the paternalistic punishment becomes all things considered beneficial only long after the punishment.

Rather than claiming that a time restriction provides a morally relevant difference between cases like Victoria and paternalistic punishment, the sophisticated retributivist could suggest a different restriction. It might be thought that a crucial difference is that Victoria’s punishment leads her to make herself all things considered better off, whereas in typical cases, it is the paternalistic punishment itself that makes the offender all things considered better off, whether the benefit occurs at the time of punishment, or later. We might think punishment that results in the offender being all things considered better off as a result of the offender’s agency is permitted by the sophisticated retributivist reason, but punishment that itself makes the offender all things considered better off is not. Let’s call this an agency/consequences restriction.

It is possible that the punishment of compelling the offender to exercise would fail to be sufficient punishment on this restriction. The health benefits received as a result of the compulsory exercise are all things considered good for the offender, without him employing his agency at all. If these benefits are greater than the harm inflicted, then the agency/consequences restriction would deem it to be an insufficient punishment for failing to give the offender what he deserves.

Even if the sophisticated retributivist accepts this restriction, there are still many forms of paternalistic punishment that it does not rule out. Suppose that we compel an offender to read a valuable piece of literature. This inflicts intentional harms on the offender.^22^ It is plausible to think that for the duration of the punishment, the harm outweighs any immediate benefit of reading. But this is not the only benefit the offender receives from the punishment. The literature might result in the offender gaining insight and understanding, and it might lead him to reflect on his life. The literacy skills enhanced by the punishment could enable him to read further works of literature, and enable him to pursue other careers and activities in the future. These are all plausible benefits of the punishment, which in the course of the offender’s life as whole make the punishment all things considered good for him. But note that these benefits are, or at least would often be, a result of his agency. So, some paternalistic punishments can make the offender all things considered better off through the agency of the offender. If the sophisticated retributivist wants to avoid the conclusion that we have not adequately inflicted punishment in cases such as Victoria by utilising the agency/ consequences restriction, this allows many paternalistic punishments to be a method of acting in accordance with sophisticated retributivist reasons.

The sophisticated retributivist might object to paternalistic punishment on different grounds. It might be thought that it is inadequate as a method of punishment simply because it fails to be a proportionate punishment. While it might be conceded that the paternalistic punishment might be sufficiently harmful when the offender deserves only minor harm, for most criminal offences it is insufficient. As a result, paternalistic punishment would be an inadequate method of acting in accordance with retributivist reasons to punish in most cases.

To a large extent this objection is understandable given the examples of paternalistic punishment provided at the start of the article. It is plausible to think that, for serious offences, the punishment of denying the offender cigarettes or requiring the offender to exercise would not inflict proportionate harm for murder or other serious offences.

One possible response would be to note that the success of my argument does not require paternalistic punishment to be proportionate as a response to all offences. I could concede that paternalistic punishment could not be proportionate retribution for the most serious offences, and traditional punishment should be employed instead in these cases. But this concession might not be necessary, as there are many types of paternalistic punishments that could inflict severe harm, while still being all things considered good for the offender. For example, as a response to sexual offences we could administer chemical castration. Suppose the offender does not want to receive the treatment, and we administer it forcibly. In addition to possible autonomy-based harms, and harms of enforcement, the treatment might also inflict considerable experiential harms, such as the harmful effects of increasing body fat, depression, hot flushes, osteoporosis, and gallstones.^23^ Yet, it is plausible to think that even if we inflict significant harms, we confer a considerable benefit to the offender, namely, we eliminate his desire to commit serious wrongdoing.

In summary, paternalistic punishment can be employed as a means of acting in accordance with crude retributivist reasons to inflict suffering on an offender, and also with sophisticated retributivist reasons to make the offender all things considered worse off, if we allow some plausible restrictions to what satisfies these reasons. I have also argued that paternalistic punishment could involve significant harm, and so can be a proportionate response to serious offences.

### 2.2 The deterrence reason to punish

Let’s now turn to a different reason to punish, namely the reason of deterrence.^24^ Broadly speaking, on this view, the reason to punish offenders is to deter further offences from being committed (either by the offender himself, or by other potential offenders). We can also act in accordance with this reason by deploying paternalistic punishment. For as long as it is sufficiently unpleasant to be subjected to paternalistic punishment, then even if being punished is all things considered good for an offender, paternalistic punishment could nonetheless be used as a deterrent.

The fact that the punishment is all things considered beneficial does not undermine its deterrent effect, for at least three reasons. The first is that people are often deterred from doing things that are unpleasant even if doing those things makes them all things considered better off. People often fail to do what is good for them if it is initially unpleasant. Consider examples such as going to the dentist, taking medicine, doing exercise, dieting, and so forth.^25^ *Ceteris paribus*, it is rational for people to prefer to incur a smaller harm now, rather than incur a larger harm later, but often people are irrational insofar as they avoid incurring smaller harms now, even when they know the avoidance results in greater harms later. Given that paternalistic punishment inflicts harm as a response to an offence, it is plausible that this would serve as a deterrent.

Second, it is important to note that even if the offender would otherwise want to perform the valuable activity or not do the harmful activity, he may not want to be compelled to do it, or be denied of it. For example, he might hold that smoking is harmful, but still not want to be forced to give up smoking. He might also hold that smoking is harmful, want to give up smoking, but not want to be forced to give up smoking. As a consequence, being forced to give up smoking could be a deterrent, even if the offender accepts the all things considered goodness of the paternalistic punishment.

The third response would be to concede that, in order to act in accordance with the deterrence reason, the paternalistic punishment needs to involve more severe harm than would be required on other theories of punishment. In itself, however, this is not an objection to paternalistic punishment, but rather it requires that we administer forms of paternalistic punishment that involve sufficiently severe harm.

There is a further objection to the view that we could act in accordance with a deterrence reason by using paternalistic punishment. It might be thought that the all things considered benefit of the punishment could create an incentive to commit an offence. For example, imagine a smoker who is struggling to give up smoking. If he knows that some offenders are punished by being denied cigarettes, this might motivate him to offend.

One response would be to note that this is contrary to the evidence cited above that states that people are often poor at doing what is all things considered good for them if it involves short term experiential harm. But even setting this to one side, it is important to note that paternalistic punishment can be individualised to ensure that it involves sufficient harm. For example, suppose one holds that paternalistic punishment is harmful because it interferes with a person’s autonomous projects and relationships. In the case of the smoker, even if it would be paternalistic to deny him tobacco with the intention to benefit him, it might not be sufficiently harmful, given that it is not contrary to his autonomous projects. Instead, in order to inflict harm sufficient to deter we might need to impose a different paternalistic punishment, one that would be contrary to his autonomous projects.

In summary, in this section I argued that, by employing paternalistic punishment, we can act in accordance with three reasons to punish, rehabilitation, retributivism, and deterrence. As noted above, this does not mean that it is permissible to do so, as there could be significant reasons not to behave paternalistically. I will turn to this point in Section 4, but before that, I set out why paternalistic punishment can be a morally preferable method of punishing criminal offenders.

## 3 The merits of paternalistic punishment

Paternalistic punishment can be morally preferable to traditional punishment in three main respects. First, paternalistic punishment might be a less harmful form of punishment, and all other things being equal, we should prefer to cause less harm than more harm. Second, any plausible criminal justice system will result in innocent people being wrongfully convicted. Given that this is the case, paternalistic punishment is preferable to traditional forms of punishment, as it is morally worse to wrong someone *and* harm them than to wrong someone in the same respect and make him all things considered better off. Third, paternalistic punishment could be a less costly way of punishing offenders, and this enables us to use resources on other valuable causes. Let me expand on each of these points in turn.

The first reason paternalistic punishment can be morally preferable is that it might be less harmful. This is due to the fact that it results in the offender being all things considered better off. While traditional, non-paternalistic forms of punishment might inflict the same amount of *pro tanto* harm as a paternalistic punishment, the latter results in the offender being all things considered better off, whereas the former typically does not.

Recall that the reason to inflict harm is an instrumental reason to achieving the end of punishment. That is, we only punish a person in order to achieve some end, such as deterrence or retribution. There is no further reason to inflict greater harm than necessary to achieve that end.^26^ In some circumstances, we can do this by inflicting *pro tanto* harms that all things considered benefit the offender, rather than simply inflicting all things considered harms as we often do when we deploy traditional, non-paternalistic forms of punishment.

For example, the crude retributivist requires a certain amount of suffering to be inflicted on the offender, and suppose we have two ways of doing this. One is a traditional, non-paternalistic punishment, the other is a paternalistic punishment. While both could satisfy the crude retributivist reason to punish because they both inflict the same amount of suffering, the paternalistic punishment would also make the offender all things considered better off, whereas the non-paternalistic punishment might not. As a result, the paternalistic punishment would be a less harmful way of acting in accordance with the crude retributivist reason to punish.

Moreover, not only is there no reason to inflict any more harm than necessary to achieve the ends of punishment, but there is a reason *not* to inflict more harm than is necessary, and doing so is a significant wrong. Recall that the reasons to punish explain why it is permissible to treat offenders in ways that it would be impermissible to treat innocent persons. Once we have satisfied the reason to punish, even offenders may not permissibly be harmed further.

The second reason paternalistic punishment can be a morally preferable means of punishing offenders is due to the fact that any plausible criminal justice system will result in the wrongful punishment of innocent people.^27^ Consider typical cases of mistaken traditional punishment, where innocent people are both wronged by being punished, and harmed by being punished. By imprisoning an innocent person, we wrong him by punishing him when he does not deserve to be punished, and we also harm him by punishing him. The punishment in many cases makes his life go all things considered worse. In contrast, consider a case where we wrongly paternalistically punish an innocent person. Of course, we still wrong the innocent person by punishing him. However, we only inflict *pro tanto* harms on him that aim to make him all things considered better off. If we are faced with a choice of wronging and all things considered harming a person, or wronging him in the same way and only *pro tanto* harming him, while actually all things considered benefitting him, the latter is morally preferable.

The third reason paternalistic punishment can be morally preferable to other forms of punishment was mentioned briefly above, namely it might result in fewer costs for the criminal justice system. If paternalistic punishment is less expensive than traditional punishment, at least in some cases, then those savings could be spent on other valuable projects. Clearly though, this would depend on the specific form of paternalistic punishment deployed, and I should be clear that my point here is tentative. I am not claiming that paternalistic punishment is necessarily cheaper than non-paternalistic punishments. Whether paternalistic punishment has a tendency to be cheaper depends on the specific type of paternalistic punishment, and the non-paternalistic alternatives. Incarceration, however, is expensive.^28^ In the UK, it costs over £40,000 a year to keep the average inmate in prison.^29^ Even paternalistic punishment that is used in conjunction with incarceration to reduce the time the offender spent incarcerated could be a significantly cheaper form of punishment. Indeed, there is evidence that paternalistic punishment could be cheaper than incarceration.^30^

## 4 The wrongness of paternalism

I have argued that paternalistic punishment can be deployed as a means of acting in accordance with three reasons to punish. I have also shown that there can, in some cases, be any or all of three advantages of administering paternalistic punishment rather than traditional forms of punishment. This does not, however, establish that it is permissible to administer paternalistic punishment. After all, paternalism is commonly thought to be wrong. If we attach sufficient weight to the wrongness of paternalism, it could account for the view that paternalistic punishment is impermissible while traditional non-paternalistic punishment is not, despite the aforementioned possible advantages of paternalistic punishment.

In order to establish that paternalistic punishment is permissible, I need to clarify what makes paternalism typically wrong. I consider two prominent views of the wrongness of paternalism: the autonomy view and the motive view. I argue that if we accept the autonomy view, and that traditional methods of punishment are permissible on the basis of at least one of the reasons to punish, we should also accept that paternalistic punishment is permissible. On the motive view, I argue that paternalistic punishment is not necessarily wrong qua paternalism at all.

According to many liberal philosophers, paternalism is wrong because it involves an interference contrary to the paternalisee’s autonomy.^31^ Suppose I prevent you from acting on an autonomous decision to eat fatty food because I believe the food is bad for your health. On this view, the paternalistic interference would be wrong because it would be contrary to your autonomous decision to eat fatty food. Let’s summarise this view as follows:

*Autonomy View:* Paternalistic behaviour is wrong qua paternalism iff there is an interference with the paternalisee’s autonomy.

If the wrongness of paternalism were autonomy-based, then the argument for the permissibility of paternalistic punishment would be straightforward. Traditional punishment interferes with an offender’s autonomy in a number of ways. When we imprison an offender we not only restrict his freedom of movement, but we frustrate the autonomous pursuit of his plans, hobbies, and relationships. It is difficult to overstate the extent to which incarceration interferes with the offender’s autonomy.

Now, if at least one of the reasons to punish is sufficient to permit interference with the offender’s autonomy by means of traditional methods of punishment, then this reason to punish should also be sufficient to permit interference with the offender’s autonomy by means of paternalistic punishment.

After all, paternalistic punishment need not be as severe a diminution of autonomy as incarceration. It only needs to interfere with one aspect of the offender’s life, whereas incarceration frustrates numerous autonomous projects and relationships. Paternalistic punishment that interferes with the offender’s autonomy would be only *pro tanto* wrong but all things considered justified by at least one of the reasons to punish.

A different account of the wrongness of paternalism is the following:

*Motive View:* Paternalistic behaviour is wrong qua paternalism iff the following two necessary and jointly sufficient conditions are met:

(1) the behaviour intends to benefit the paternalisee
(2) the behaviour is motivated by a negative judgement of the beneficiary’s ability (assuming he has the relevant information) to make the right decision or manage the particular situation, in a way that will effectively advance his wellbeing in some respect.^32^

On this view, when I interfere with your decision to eat fatty foods with the intention to benefit you, while being motivated by a negative judgement about your ability to eat a balanced diet, this is wrong qua paternalism, but if I did the same with the intention to reduce the effects of eating fatty food on the environment, it is not wrong qua paternalism.

This view might seem to pose a problem for the permissibility of paternalistic punishment. This is because even if traditional methods of punishment involve significant restrictions of autonomy, they would not be wrong qua paternalism. Paternalistic punishment, however, at least prima facie involves the objectionable intentions and motives towards the offender that make such behaviour wrong qua paternalism. This would mean paternalistic punishment is morally problematic in a respect that traditional punishment is not. If we attach sufficient weight to the wrongness of paternalism, paternalistic punishment could be impermissible.

Now, it might be thought that all punishment is motivated by a negative judgement of the offender’s ability to some extent. After all, it is deployed in response to his wrongdoing, while expressing disapproval of that wrongdoing. In which case, why think paternalistic punishment is any more difficult to justify than other punishment? On the motive view, however, there is something particularly insulting about acting from a negative judgement of a person’s ability to promote his own good, compared with acting from a negative judgement of his treatment of others. This could be because the ability to effectively advance one’s own wellbeing is more fundamental for practical reasoning than that which concerns the treatment of others. Or it could be because one’s own interests are legitimately one’s own domain, and a failure to respect this domain is insulting.^33^ Regardless of the precise basis for this distinction, the special insult of paternalism could be held to be sufficiently great to render paternalistic punishment impermissible.

While this objection has traction in some cases of paternalistic punishment, in many it does not. First of all, suppose when considering the possible equally effective punishments that would satisfy one of the reasons to punish, we opt for the one that is the cheapest. Suppose it just so happens that the cheapest punishment is one that benefits an offender all things considered. In such cases, there is not necessarily any objectionable paternalistic intention and motive. We choose the punishment that benefits the person all things considered not because we intend to benefit the offender, or because we are motivated by a negative judgement, but rather, we choose it to reduce costs for the criminal justice system. When we do this, it would not be wrong qua paternalism, and so would not be a method of punishment objectionable in a respect that non-paternalistic punishment is not. But as noted in Section 3, whether it will cost less depends on the specific type of paternalistic punishment administered, and we can assume that not all will be cheaper.

Instead, consider a more challenging case. Suppose of all the possible equally effective punishments of the same cost, we opt for the one that is the least harmful for the offender. It might be thought that one does not have the relevant paternalistic intentions and motives when one chooses the least harmful form of punishment. Rather, it is simply more humane to impose a less harsh punishment on someone. This thought, however, is mistaken. When we choose paternalistic punishment rather than traditional punishment on the basis that it is less harmful, in many cases we are required to hold both an intention to benefit the offender, and a negative judgement of the offender’s abilities to effectively advance his wellbeing. For instance, in order to believe that compelling the offender to give up smoking is less harmful, we must hold a negative judgement concerning the offender’s smoking. It is the fact that smoking is bad for the offender that makes preventing him from smoking all things considered beneficial, and so, a less harmful punishment. But by holding this view of his actions, we seem to be motivated by a negative judgement of his abilities to effectively advance his wellbeing. The intention to benefit the offender can be seen by the fact that we are choosing to administer a paternalistic punishment rather than a non-paternalistic punishment because it makes the offender all things considered better off.

In response, we should concede that *some* cases of paternalistic punishment require objectionable paternalistic motives and intentions, but many do not. Recall the distinction between two types of paternalistic punishment. The first type denies the offender something bad for him, such as tobacco. The second type of paternalistic punishment compels the offender to do something that benefits him, such as reading valuable literature. While objectionable paternalistic motives and intentions are required in the former type of paternalistic punishment, this is not the case in the latter type of paternalistic punishment. A person can be compelled to do something that is good for him, without holding any negative judgement about his abilities to effectively advance his wellbeing.

This is because even if the offender has never read valuable literature before, compelling him to read it does not necessarily mean his life is going badly in any respect. We could hold value pluralism to be true.^34^ This is the view that there are ‘various forms and styles of life which exemplify different virtues and which are incompatible’.^35^ For example, I have never gone horse riding, and because it is a valuable activity, it would be good for me to go horse riding. But it does not follow that my life is worse in any respect for spending my time playing cricket rather than riding horses. There is a plurality of valuable pursuits that would be good for me to do, but my life is not necessarily going worse for not having done them all.^36^

With regard to punishment, we know that we ought to inflict the least harmful of all effective punishments that satisfy the reason to punish, and compelling a person to do something valuable is one method of doing this. We can inflict the requisite amount of *pro tanto* harm to satisfy the reason to punish by compelling a person to do something valuable. But this does not require a negative judgement of the offender’s abilities to effectively advance his wellbeing. It just requires us to know that the offender would receive the required amount of harm, and that the activity would be all things considered good for him.

It might be objected that this argument proves too much. Does it entail that on the motive view, any paternalistic compulsion would be permitted, even towards nonoffenders? After all, couldn’t a paternaliser also appeal to a plurality of valuable pursuits in order to claim that when he compels a paternalisee to do any valuable activity he is not motivated by a negative judgement of the paternalisee?

The reason this argument does not have this implication is that there is a crucial difference between the offender and the non-offender. We can see this if we ask, why compel them to do the valuable activity? In the case of the offender there is a convincing answer, namely, that we do so in order to satisfy one of the reasons to punish. By being compelled, the offender receives the required amount of harm, which is all things considered good for him. In the case of the non-offender, we cannot appeal to these reasons, as reasons to punish do not apply to non-offenders. Instead, we must appeal to the fact that the paternalisee would be better off if we compelled him to do the activity. But this entails that we are motivated by a negative judgement. It means we judge that he is less likely to do the activity without our compulsion, or that he is making a mistake by not doing it. As a result, even if the paternaliser believes value pluralism to be true, it does not permit paternalistic compulsion in cases of non-offenders.

In summary, the wrongness of paternalism does not mean paternalistic punishment is impermissible. I considered the autonomy view, and argued that if at least one of the reasons to punish is sufficient to permit interference with the offender’s autonomy by means of traditional methods of punishment, then this reason to punish should also be sufficient to permit interference with the offender’s autonomy by means of paternalistic punishment. Alternatively, on the motive view, we can administer all forms of paternalistic punishment without it being wrong qua paternalism when it is the cheapest effective punishment. We can also compel the offender to do activities that benefit him without it being wrong qua paternalism on the motive view when the paternalistic punishment is of an equal cost to other effective punishments.

## 5 Conclusion

I have argued that even if paternalistic behaviour is impermissible towards innocent persons, the same paternalistic behaviour can be permissible when directed towards offenders. It can be permissible on the same grounds that inflicting intentional harm is permissible, namely that it is sometimes a means of acting in accordance with three reasons to punish, namely, rehabilitation, retributivism, and deterrence. I argued that paternalistic punishment can also be a morally preferable form of punishment. It can be less harmful, preferable given the risk of mistakenly punishing innocent persons, and a less costly method of punishment. I then argued that even if one holds paternalistic behaviour to be significantly wrong, this does not necessarily mean that paternalistic punishment is impermissible.^37^

## Footnotes

1 Henceforth I refer to criminal offenders as offenders, for short.

2 These examples are intended to be merely illustrative rather than concrete proposals for alternative punishments. However, some of these activities are already provided on a voluntary basis for offenders. See the Cebu Provincial Detention Center in the Philippines, and the Changing Lives Through Literature Program in the USA. For a discussion of the latter see Robert P. Waxler, Changing Lives through Literature, *PMLA*, 123 (2008), 678-82.

3 By making this claim, I assume that there is no requirement that the punishment should be qualitatively proportionate – the idea that ‘the action which constitutes punishment for an offense should possess some or all of the characteristics that made the offense wrong’. Jeremy Waldron, Lex Talionis, *Arizona Law Review*, 34 (1992), 26–51 (p. 35). If one holds this requirement, then it limits the types of paternalistic punishment that could be permissibly administered.

4 Plato, *Gorgias*, 476a–479e; Soren Kierkegaard, *The Sickness Unto Death* (London: Penguin, 2008), p. 37; John M. E. McTaggart, Hegel’s Theory of Punishment, *International Journal of Ethics*, 6 (1896), 479–502.

5 Jean Hampton, The Moral Education Theory of Punishment, *Philosophy and Public Affairs*, 13 (1984), 208–38; Herbert Morris, A Paternalistic Theory of Punishment, *American Philosophical Quarterly*, 18 (1981), 263–71.

6 See David Boonin, *The Problem of Punishment* (Cambridge: Cambridge University Press, 2008), pp. 180–91.

7 This definition is adapted from Jonathan Quong, *Liberalism Without Perfection* (Oxford: Oxford University Press, 2011), p. 80. My argument is successful regardless of the definition of paternalism one holds.

8 This definition is adapted from Boonin, pp. 24–25. My argument would be successful on any of the major definitions of punishment. For the sake of elegance, henceforth I omit ‘alleged’.

9 H. L. A. Hart, The Presidential Address: Prolegomenon to the Principles of Punishment, *Proceedings of the Aristotelian Society*, 60 (1959), 1–26.

10 R. A. Duff, *Punishment, Communication, and Community* (Oxford: Oxford University Press, 2001).

11 Thom Brooks, *Punishment* (London: Routledge, 2012), p. 2.

12 John Finnis, Retribution: Punishment’s Formative Aim, *American Journal of Jurisprudence*, 44 (1999), 91–103 (p. 98).

13 I use this example to make the same point in David Birks, Can Neurointerventions Communicate Censure? (And So What If They Can’t?), in *Treatment For Crime: Philosophical Essays on Neurointerventions in Criminal Justice*, ed. by David Birks and Thomas Douglas (Oxford: Oxford University Press, 2018), pp. 351–68 (p. 359).

14 Michael Bratman, *Intention, Plans, and Practical Reason* (Cambridge, MA: Harvard University Press, 1987), p. 29.

15 Joel Feinberg, The Expressive Function of Punishment, in *Doing and Deserving: Essays in the Theory of Responsibility* (Princeton: Princeton University Press, 1970), pp. 95–118.

16 For England and Wales, see Re T (Adult: Refusal of Medical Treatment) [1992] 4 All ER 649.

17 See: Jens Modvig, Violence, Sexual Abuse and Torture in Prisons, in *Prisons and Health*, ed. by Stefan Enggist, Lars Møller, Gauden Galea, and Caroline Udesen (Copenhagen: World Health Organization), pp. 19–26.

18 Peter Raynor and Gwen Robinson, Why Help Offenders? Arguments For Rehabilitation as a Penal Strategy, *European Journal of Probation*, 1 (2009), 3–20.

19 For a similar formulation, see Victor Tadros, *The Ends of Harm: The Moral Foundations of Criminal Law* (Oxford: Oxford University Press, 2012), p. 26.

20 Although proponents of retributivism do not explicitly present their view in these terms, I think it is the most plausible interpretation. See, for instance, Michael S. Moore, *Placing Blame: A Theory of the Criminal Law* (Oxford: Oxford University Press, 2010).

21 NHS, *Benefits of Exercise* (2018) <https://www.nhs.uk/live-well/exercise/exercise-health-benefits/> [accessed 24 July 2020].

22 These harms might include a harm to the offender’s autonomy by requiring him to read the literature, as well as possible experiential harms of frustration and boredom, as well as the possible harms necessary to ensure the offender has read the literature.

23 Peer Briken and Martin P. Kafka, Pharmacological Treatments for Paraphilic Patients and Sexual Offenders, *Current Opinion in Psychiatry*, 20 (2007), 609–13.

24 See for example: Antony Ellis, A Deterrence Theory of Punishment, *The Philosophical Quarterly*, 53 (2003), 337–51.

25 For example, in the USA, despite the fact the average cost of gym membership is $58 per month, 67% of people with gym membership never attend their gym. Statistics Brain Research Institute, *Gym Membership Statistics*, (2016) <https://www.statisticbrain.com/gym-membership-statistics/> [accessed 5 April 2017].

26 For a discussion of this point, see Tadros, p. 103.

27 Tadros (p. 105) also notes that punishing innocent persons is an inevitable harm of a workable criminal justice system.

28 Christian Henrichson and Ruth Delaney, *The Price of Prisons: What Incarceration Costs Taxpayers* (New York: Vera Institute of Justice, 2012), p. 6.

29 Ministry of Justice, *Costs Per Place and Costs Per Prisoner by Individual Prison, National Offender Management Service Annual Report and Accounts 2010–11* (London: Ministry of Justice, 2011), p. 4.

30 For the financial advantages of requiring offenders to read valuable works of literature as an alternative to incarceration, see G. Roger Jarjoura and Susan T. Krumholz, Combining Bibliotherapy and Positive Role Modeling as an Alternative to Incarceration, *Journal of Offender Rehabilitation*, 28 (1998), 127–39.

31 See, for instance, Joel Feinberg, *The Moral Limits of the Criminal Law: Harm to Self* (Oxford: Oxford University Press, 1986).

32 This view is adapted from Quong, pp. 80–102. For a different account that grounds its wrongness on the objectionable motives of the paternaliser, see Seana Valentine Shiffrin, Paternalism, Unconscionability Doctrine, and Accommodation, *Philosophy and Public Affairs* 29 (2000), 205–50 (p. 220).

33 For a critical discussion of this distinction see: Peter De Marneffe, Avoiding Paternalism, *Philosophy & Public Affairs*, 34 (2006), 68–94 (pp. 77–80).

34 Importantly, it does not matter whether value pluralism is in fact true. What matters is whether the paternaliser holds it to be true, given that we are concerned with whether the paternaliser is motivated by a negative judgement of the paternalisee.

35 Joseph Raz, *The Morality of Freedom* (Oxford: Clarendon Press, 1986), p. 395.

36 For this view, see, Joseph Raz, The Role of Well-Being, *Philosophical Perspectives*, 18 (2004), 269–94.

37 I thank Brendan Abernathy, Paul Billingham, Paul Bou-Habib, Ian Carroll, Roger Crisp, Tom Douglas, Antony Duff, Melissa Fahmy, Jeff Howard, Doug Husak, Sam Kiss, Rune Klingenberg, Judith Lichtenberg, Thomas Søbirk Petersen, Jonathan Quong, Mona Rudolf, Jesper Ryberg, Tom Sinclair, Hillel Steiner, Tony Taylor, Frej Klem Thomsen, and Stephen White for their feedback on the article. I presented earlier versions at the Workshop in Applied Ethics and Public Policy, Bowling Green State University, the Rocky Mountain Ethics Congress, University of Colorado Boulder, the Society for Applied Philosophy Annual Conference, University of Edinburgh, the Philosophy Colloquia, University of Georgia, the Overcriminalization and Indigent Legal Care Conference, Georgia State University, the MANCEPT Workshops in Political Theory, University of Manchester, the Braga Meetings in Ethics and Political Philosophy, University of Minho, the Research Group for Criminal Justice Ethics, Roskilde University, and the Fighting for Prison Reform Roundtable Workshop, University College London. I thank the participants of these events for their comments and questions. I am also grateful to the editor, and the anonymous reviewers for their helpful suggestions and objections. The article was written with the help of funding from the Wellcome Trust (100705/Z/12/Z) and the German Ministry of Education and Research (01GP1311).

